# Supplementary material for: Suppression of autophagic activity by Rubicon is a signature of aging
Source: Nat Commun. 2019 Feb 19;10:847. doi: 10.1038/s41467-019-08729-6 (PMC6381146; doi:10.1038/s41467-019-08729-6)
Supplement: Supplementary file 1 — Supplementary information [file 41467_2019_8729_MOESM1_ESM.pdf]

Nakamura, S et al.: Suppression of autophagic activity by Rubicon is a signature of aging

Supplementary Materials: 10 Figures; 2 Tables

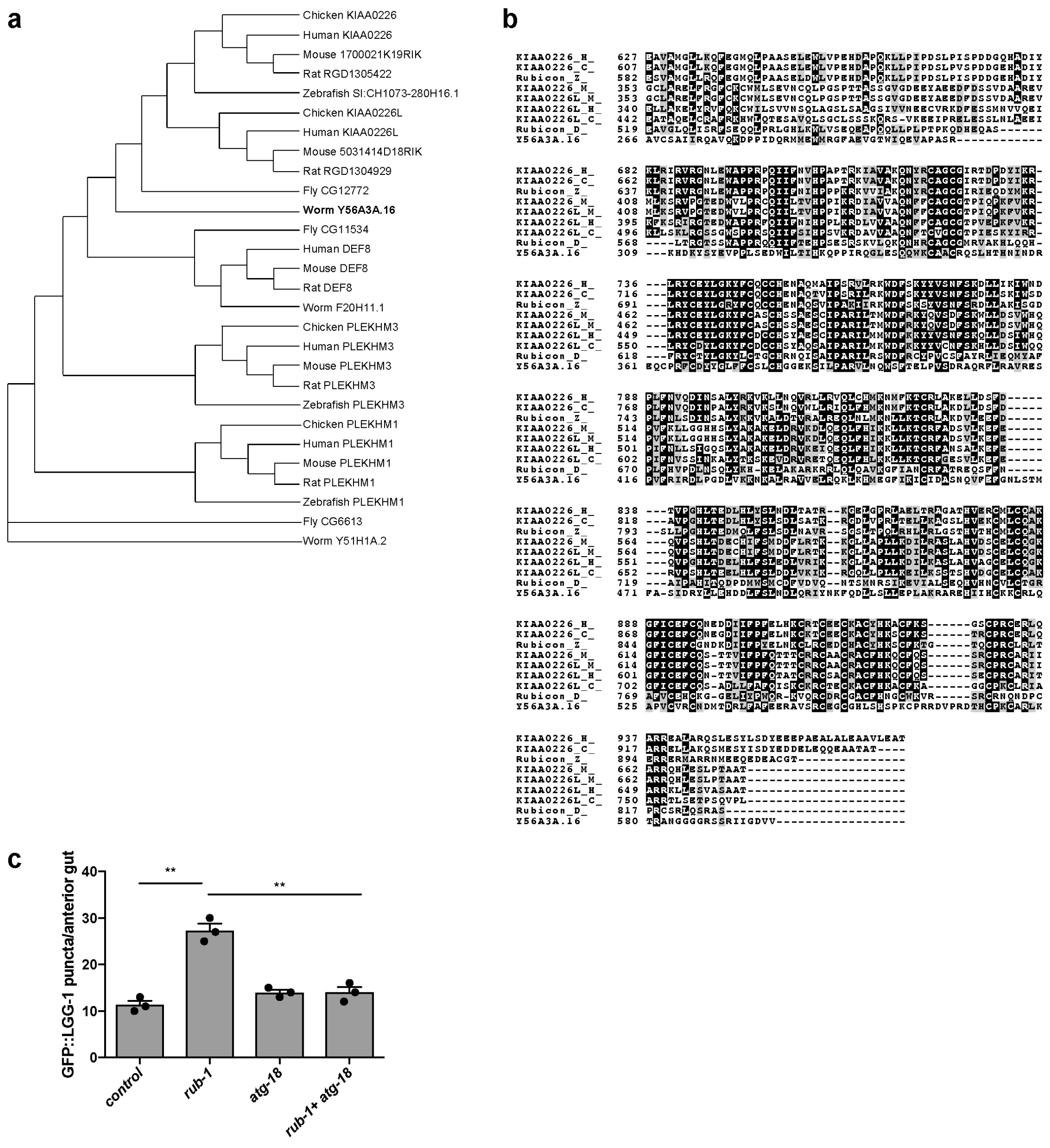

Supplementary Figure 1: Identification of *C. elegans* Rubicon homolog

(a) Phylogenetic tree generated by ClustalW analysis showing that Y56A3A.16 is similar to human KIAA00226 (Rubicon), human KIAA0226L, and *Drosophila* Rubicon(CG12772). (b) Amino-acid alignment of the C-terminal regions of Rubicon homologs from several species (H, human; C, chicken; Z, zebrafish; M, mouse; D, *Drosophila*). (c) Quantification of GFP::LGG-1 puncta in anterior intestines under each knockdown condition at L4 stage. Values represent means ± s.e.m. (n=3). *P* value (** *P*<0.01) was determined by one-way ANOVA with Tukey's test.

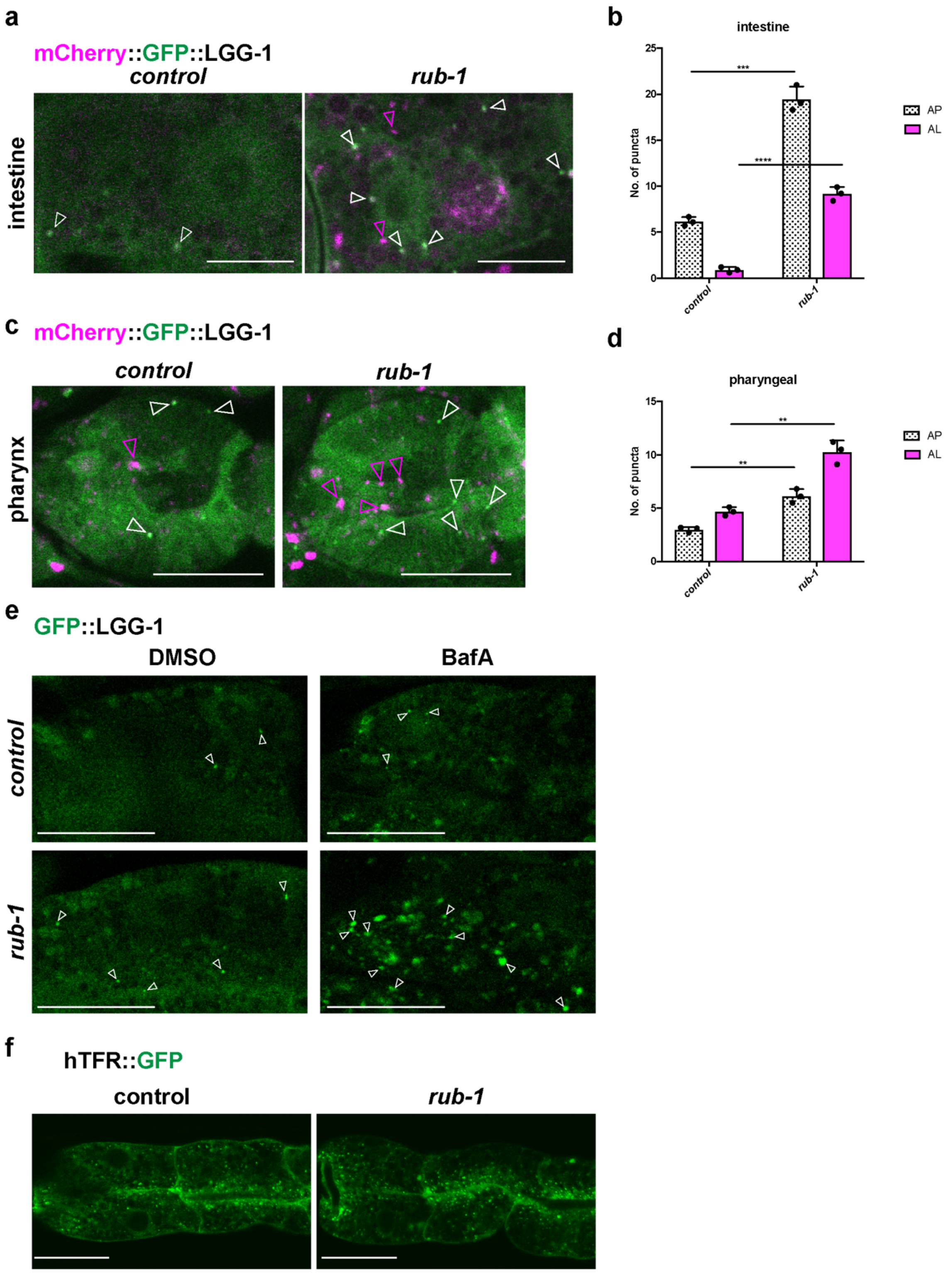

Supplementary Figure 2: autophagic activity is increased by *rub-1* knockdown

(a-d) The pool size of autophagosomes (AP) and autolysosomes (AL) was increased by *rub-1* knockdown both in intestine (a, b) and pharynx (c, d). The representative pictures

of intestine (a) and pharyngeal muscle (c) in MAH215(mCherry::GFP::LGG-1) strain subjected to control or *rub-1* RNAi at day1 adult stage. Both autophagosomes (AP, GFP+ mCherry+, indicated open white arrowheads) and autolysosomes (AL, GFP- mCherry+, indicated by open magenta arrowheads) were increased by *rub-1* knockdown. Quantification of AP and AL in intestine (b) and pharynx (d). Values represent means ± s.e.m. (n=3). *P* value (***P*<0.01, ****P*<0.001, *****P*<0.0001) was determined by *t*-test. Knockdown was conducted from egg onward. (e) The representative pictures of GFP::LGG-1 transgenic worms subjected to control and *rub-1* RNAi at day1 adult stage. Knockdown was conducted from egg onward. The pictures were obtained 2hrs after DMSO or Bafilomycin A1 microinjection. Arrowheads indicate GFP::LGG-1 puncta. (f) The localization of hTFR::GFP puncta in intestine was largely unaffected by *rub-1* knockdown at day1 adult stage. Knockdown was conducted from egg onward. Scale bars, 10 µm (a, c); 20µm (e, f).

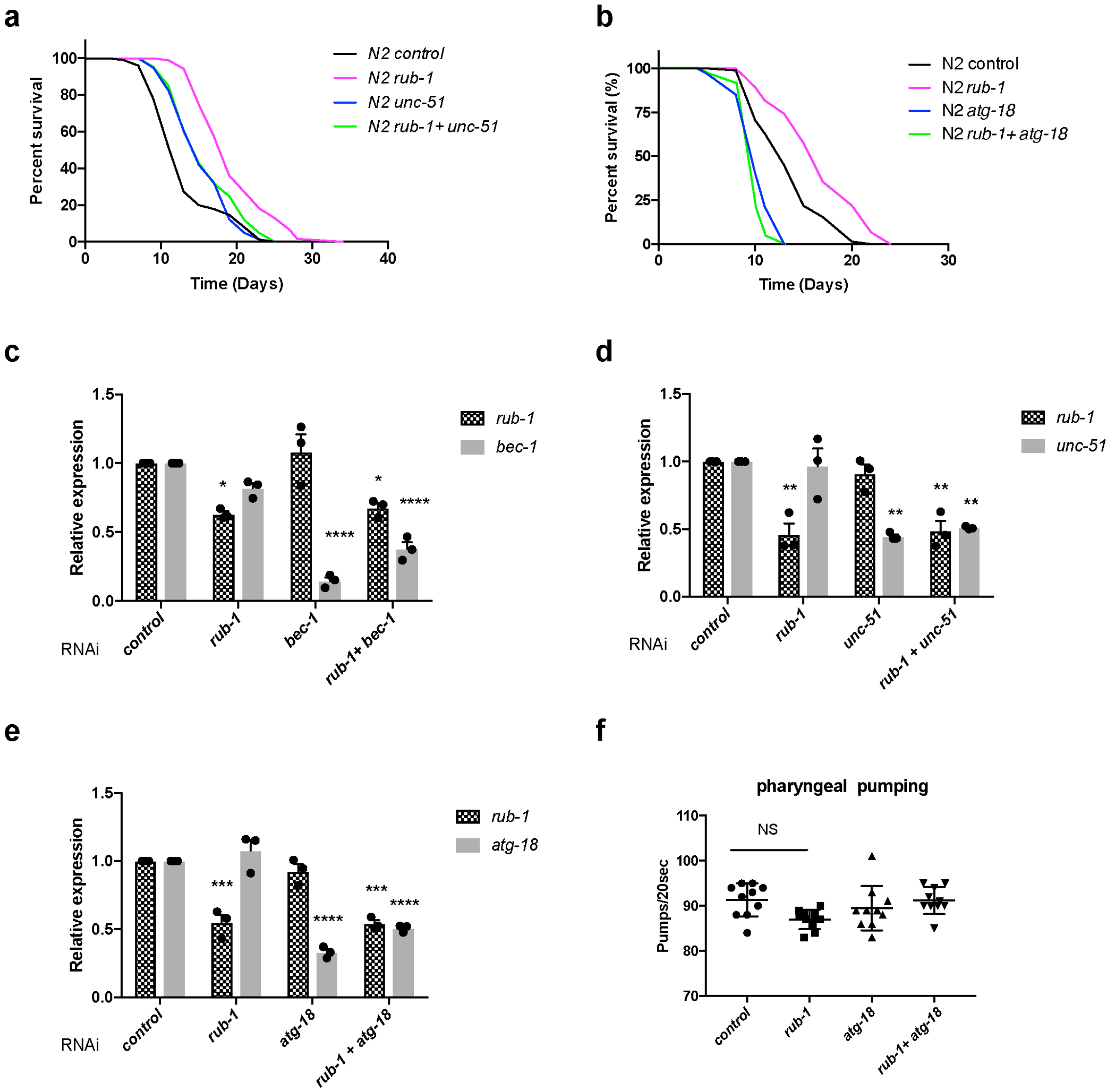

Supplementary Figure 3: *rub-1* knockdown extends lifespan

(a, b) *rub-1* knockdown extended lifespan, but concomitant knockdown of *unc-51/ULK1*(a) or *atg-18/WIPI*(b) abolished the longevity. (c-e) qRT-PCR analysis showing knockdown efficiency of *rub-1*, *bec-1*, *unc-51* and *atg-18* by RNAi in N2 background at day1 adult stage. Knockdown was conducted from egg onward. Mean ± s.e.m. from three independent experiments are depicted relative to wild type N2 with control RNAi. *P* value (* *P*<0.05, ***P*<0.01, *** *P*<0.001, *****P*<0.001) was determined by one-way ANOVA with Tukey's multiple comparisons test. (f) The pharyngeal pumping rate was not altered in *rub-1* knockdown at day1. Knockdown was conducted from egg onward. Values represent means ± s.e.m. (n=3).

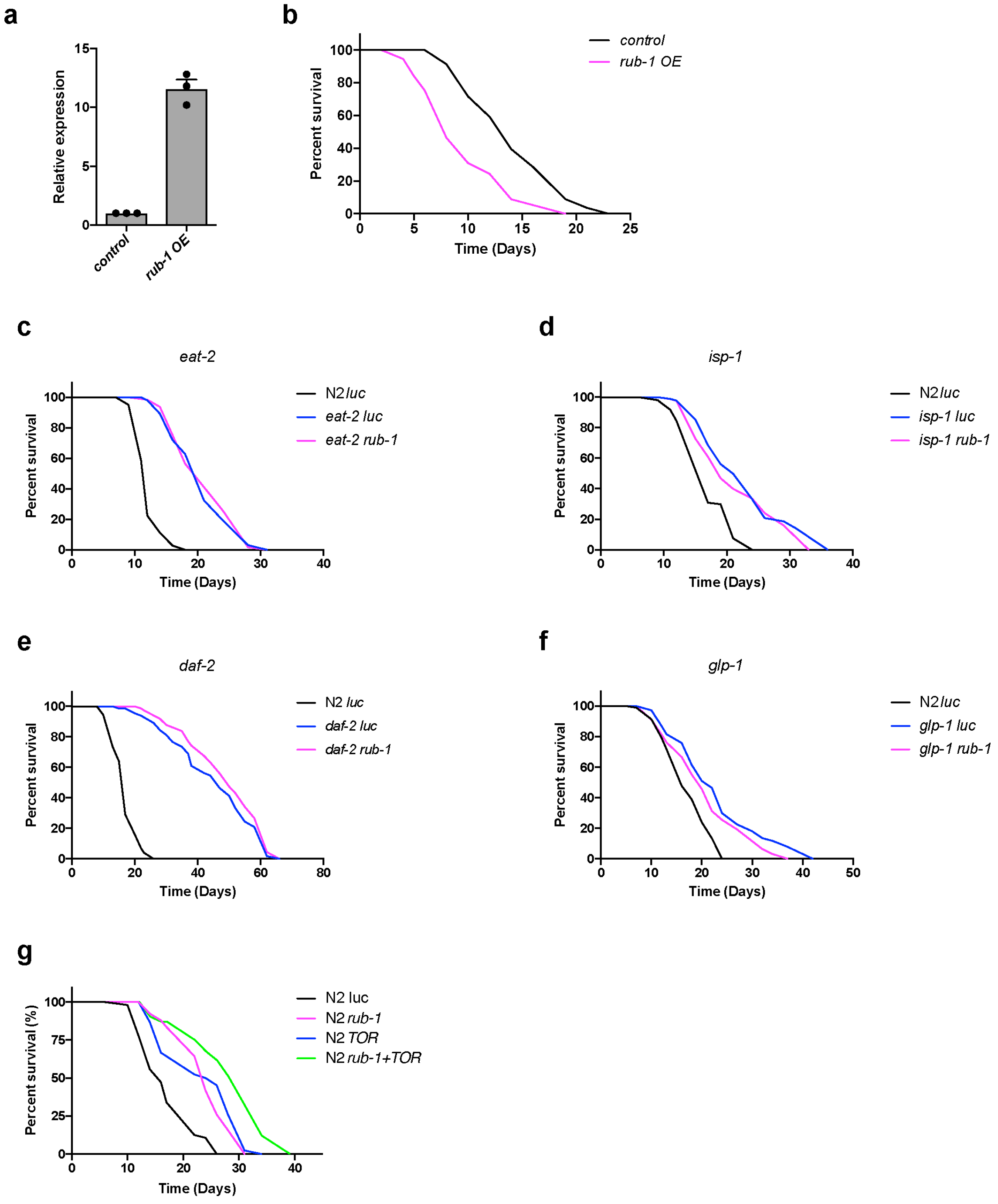

Supplementary Figure 4: Genetic interaction between *rub-1* and several longevity pathways

(a) qRT-PCR confirmed that the transgenic worm used in (b) shows the increased *rub-1* expression. Values represent means ± s.e.m. (n=3). (b) Overexpression of *rub-1* shortened lifespan of wild type worms. (c-g) Genetic interaction between *rub-1* and other longevity pathways. *rub-1* knockdown did not further extend the longevity of *eat-2* (calorie restriction), *isp-1*(mitochondrial dysfunction), *daf-2* (reduced Insulin/IGF-1 signaling)

and *glp-1*(germline longevity). Interestingly *rub-1* knockdown further extended TOR knockdown mediated longevity (i). Knockdown was conducted from egg onward for *daf-2*, *isp-1*, *eat-2* and *glp-1* lifespan experiments, while knockdown was carried out during adult only for TOR knockdown lifespan. See Supplementary Data 1 for details and repeats.

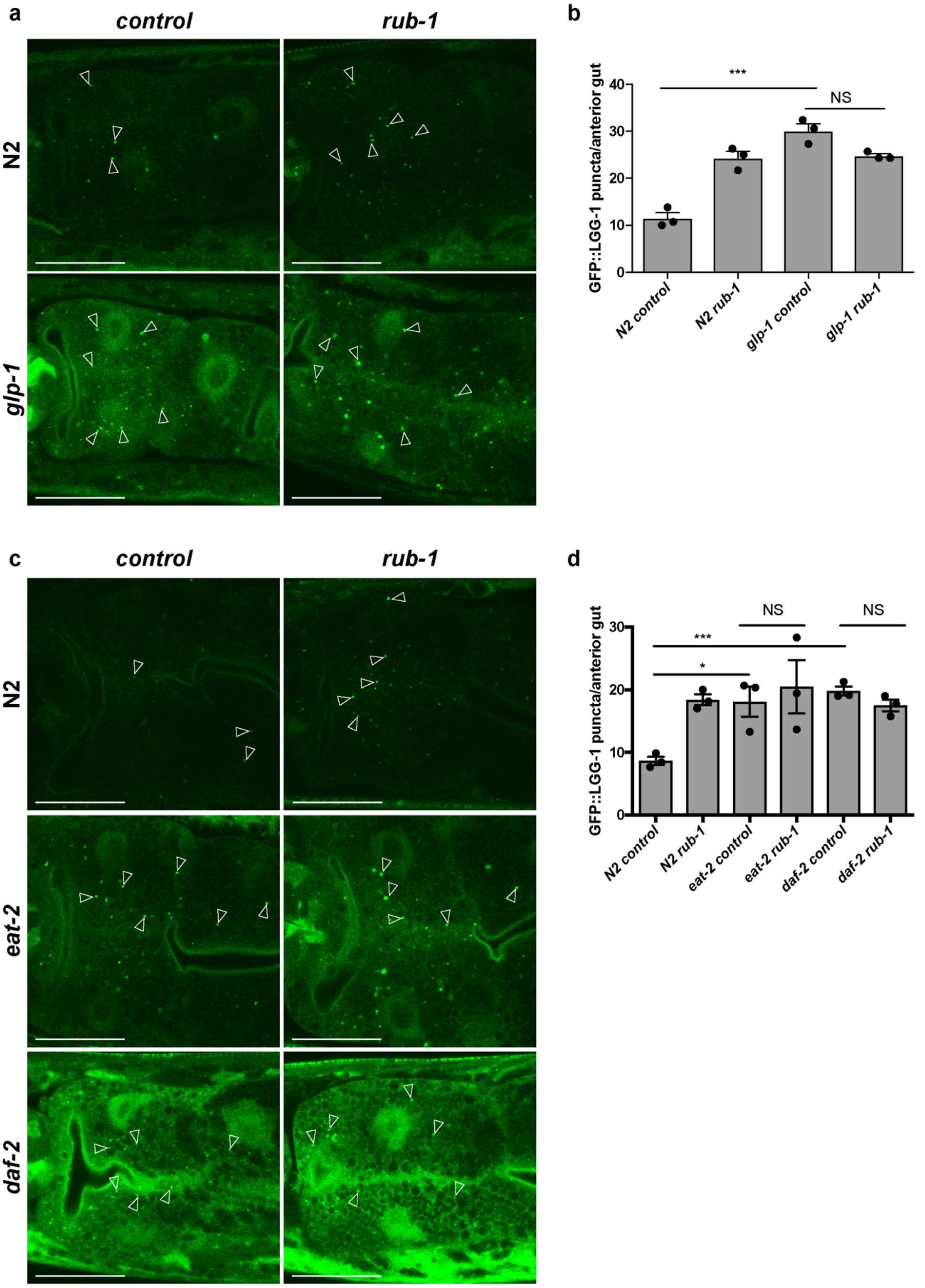

Supplementary Figure 5: *rub-1* knockdown does not further increase the numbers of autophagosomes in long-lived animals

(a) GFP::LGG-1 puncta (open arrow heads) of intestines subjected to *control* or *rub-1*

RNAi knockdown in N2 or *glp-1(e2141)* background at day1. Numbers of GFP::LGG-1 puncta were not significantly altered by *rub-1* knockdown compared to control knockdown in *glp-1* background. Knockdown was conducted from egg onward. (b) Quantification of GFP::LGG-1 puncta in anterior intestines under each knockdown condition in (a). Values represent means ± s.e.m. (n=3). *P* value (*** *P*<0.001) was determined by one-way ANOVA with Tukey's test. (c) GFP::LGG-1 puncta (open arrow heads) of intestines subjected to *control* or *rub-1* RNAi knockdown in N2, *eat-2(ad465)* or *daf-2(e1370)* background at day1. Knockdown was conducted from egg onward Numbers of GFP::LGG-1 were not significantly altered by *rub-1* knockdown compared to control knockdown in *eat-2* and *daf-2* background. (d) Quantification of GFP::LGG-1 puncta in anterior intestines under each knockdown condition in (c). Values represent means ± s.e.m. (n=3). *P* value (* *P*<0.05, *** *P*<0.001) was determined by one-way ANOVA with Tukey's test. For the technical reason, the cross between GFP::LGG-1 and *isp-1* (qm150) did not work in our hands. Thus, we could not assess the autophagic activity in *isp-1* background after *rub-1* knockdown. Scale bars, 20 μm (a, c).

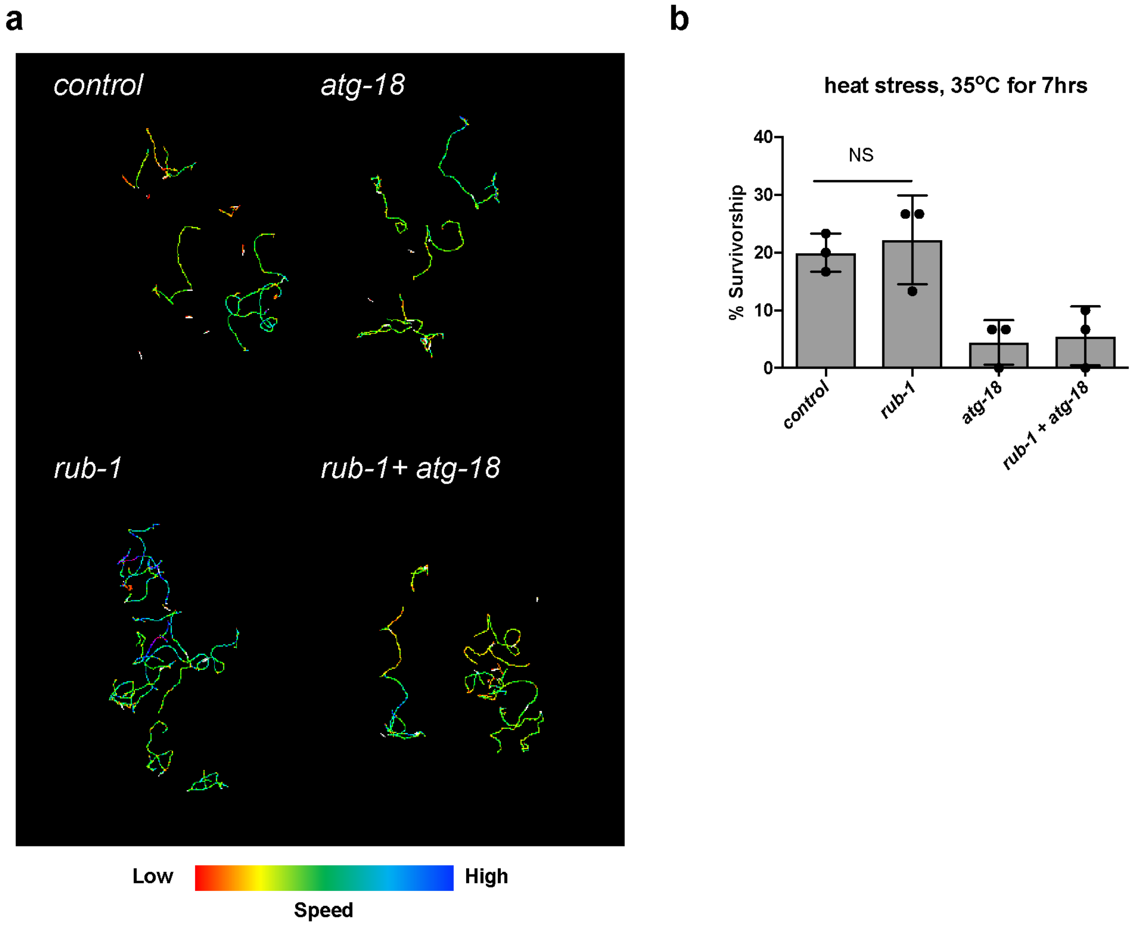

Supplementary Figure 6: Phenotypic characterization of *rub-1* knockdown worms

(a) Snapshot of multi-worm tracking analysis. Track and speed (high speed, blue; low speed, red) of individual worms were visualised under the indicated RNAi treatments. A quantification is shown in Fig. 2c. (b) Heat stress resistance assay revealed that *rub-1* knockdown did not change the resistance. Values represent means ± s.e.m. (n=3). Knockdown was conducted from egg onward.

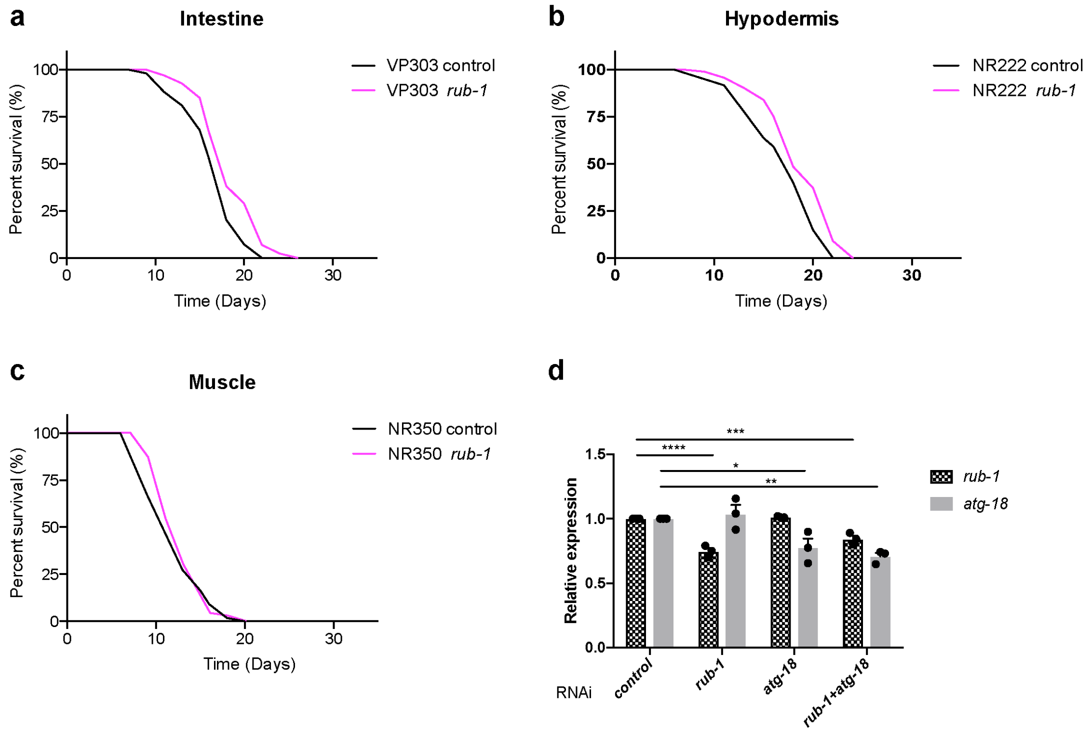

Supplementary Figure 7: Tissue specific roles of Rubicon contributing to lifespan regulation

(a-c) Demographic analysis using several tissue-specific RNAi-sensitive strains such as muscle(NR350), Hypodermis (NR222) and intestine(VP303). Knockdown was conducted from egg onward. Intestinal and hypodermal *rub-1* knockdown slightly but significantly extended lifespan. (d) qRT-PCR analysis showing knockdown efficiency of *rub-1* and *atg-18* by RNAi in TU3401(neuron specific RNAi sensitive strain) background. Mean ± s.e.m. from three independent experiments are depicted relative to TU3401 with control RNAi. *P* value (* $P<0.05$, **$P<0.01$, ***$P<0.001$, ****$P<0.001$) was determined by one-way ANOVA with Tukey's test.

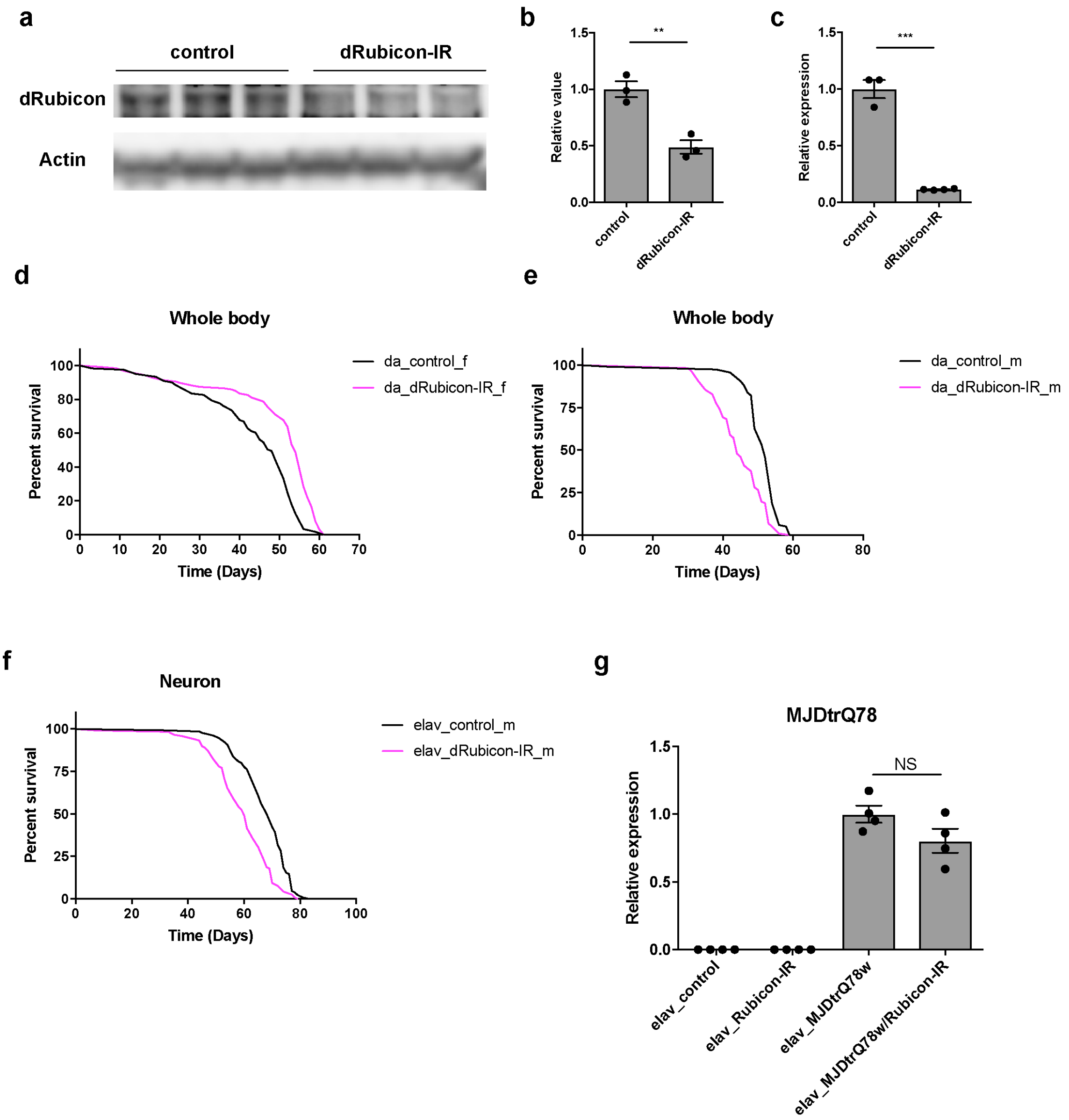

Supplementary Figure 8: dRubicon knockdown modestly extends lifespan in female fly

(a) Newly developed dRubicon antibody detected endogenous dRubicon. Note that knockdown of dRubicon decreased the band intensity. (b) A quantification of dRubicon normalized by Actin in (a). dRubicon was significantly downregulated. Values represent means ± s.e.m. (n=3). *P* value (**P<0.01) was determined by t-test. (c) qRT-PCR analysis showing that *dRubicon* was significantly downregulated by the knockdown in female fly. *P* value (**** *P*<0.0001) was determined by *t*-test (control, n=3; dRubicon-IR, n=4). (d, e) The demographic analysis of female (d) and male(e) fly subjected to whole body knockdown of control or dRubicon. dRubicon knockdown modestly but significantly

extended lifespan in female fly. See Supplementary Data 1 for statistics. (f) The demographic analysis of male fly subjected to neuron specific knockdown of control or dRubicon. (g) qRT-PCR analysis of strains used for climbing assay revealed that MJDtrQ78 transgene expression was not altered between control and dRubicon knockdown. Values represent means ± s.e.m. (n=4).

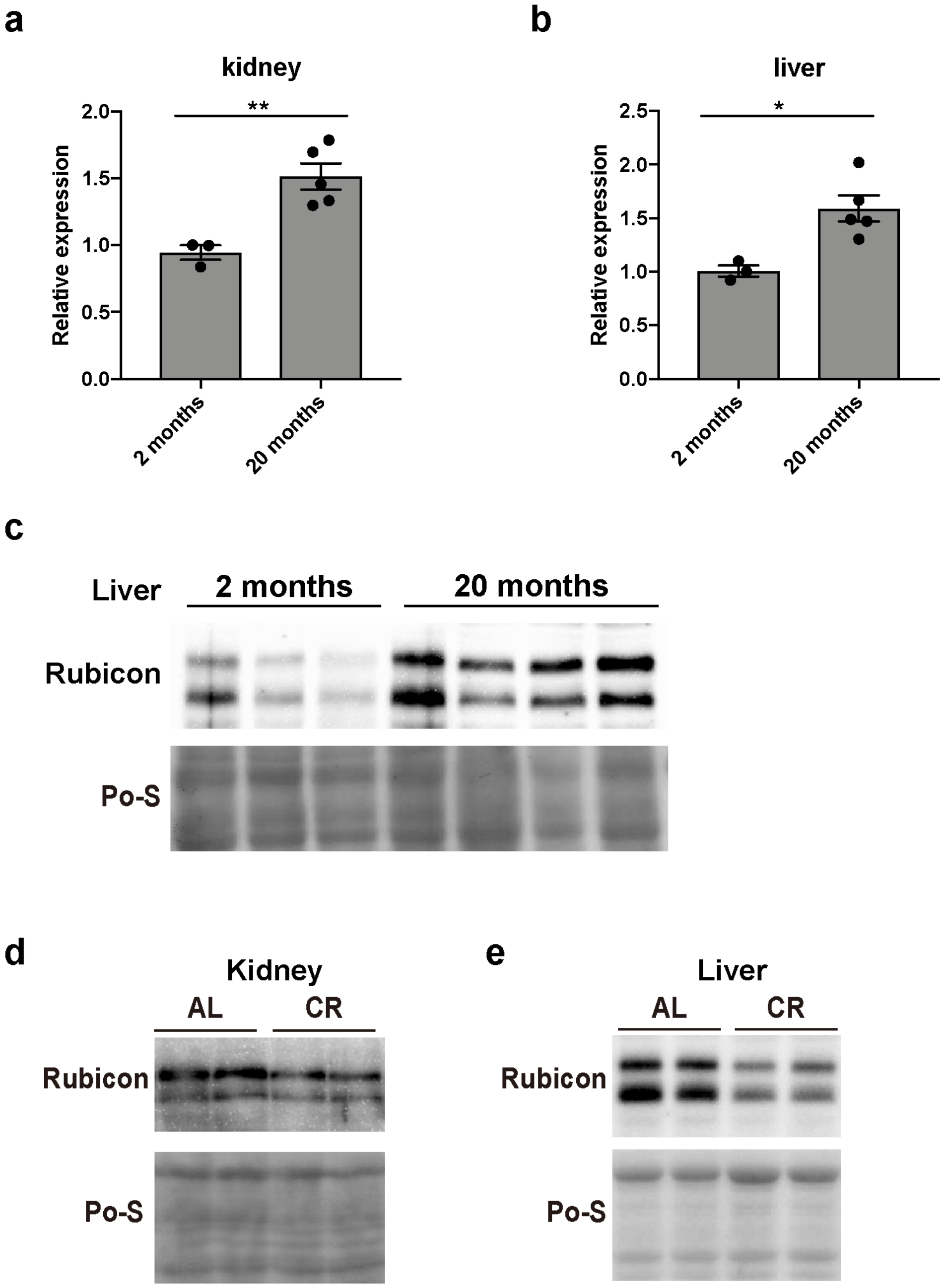

Supplementary Figure 9: Rubicon is reduced by calorie restriction in mice

(a, b) qRT-PCR analysis showing that *Rubicon* was upregulated during aging (2 months vs 20 months) in mouse kidney (a) and liver (b). The values represent the mean ± s.e.m. *P* values (** P<0.01) were determined by t-test (n=3 for 2 months, n=5 for 20 months). (c) Rubicon protein levels were increased with age (2 months vs 20 months) in mouse liver. (d, e) Nine-month calorie restriction (CR) decreased Rubicon protein levels in kidney(d) and liver (e) compared to ad libitum (AL) feeding, respectively.

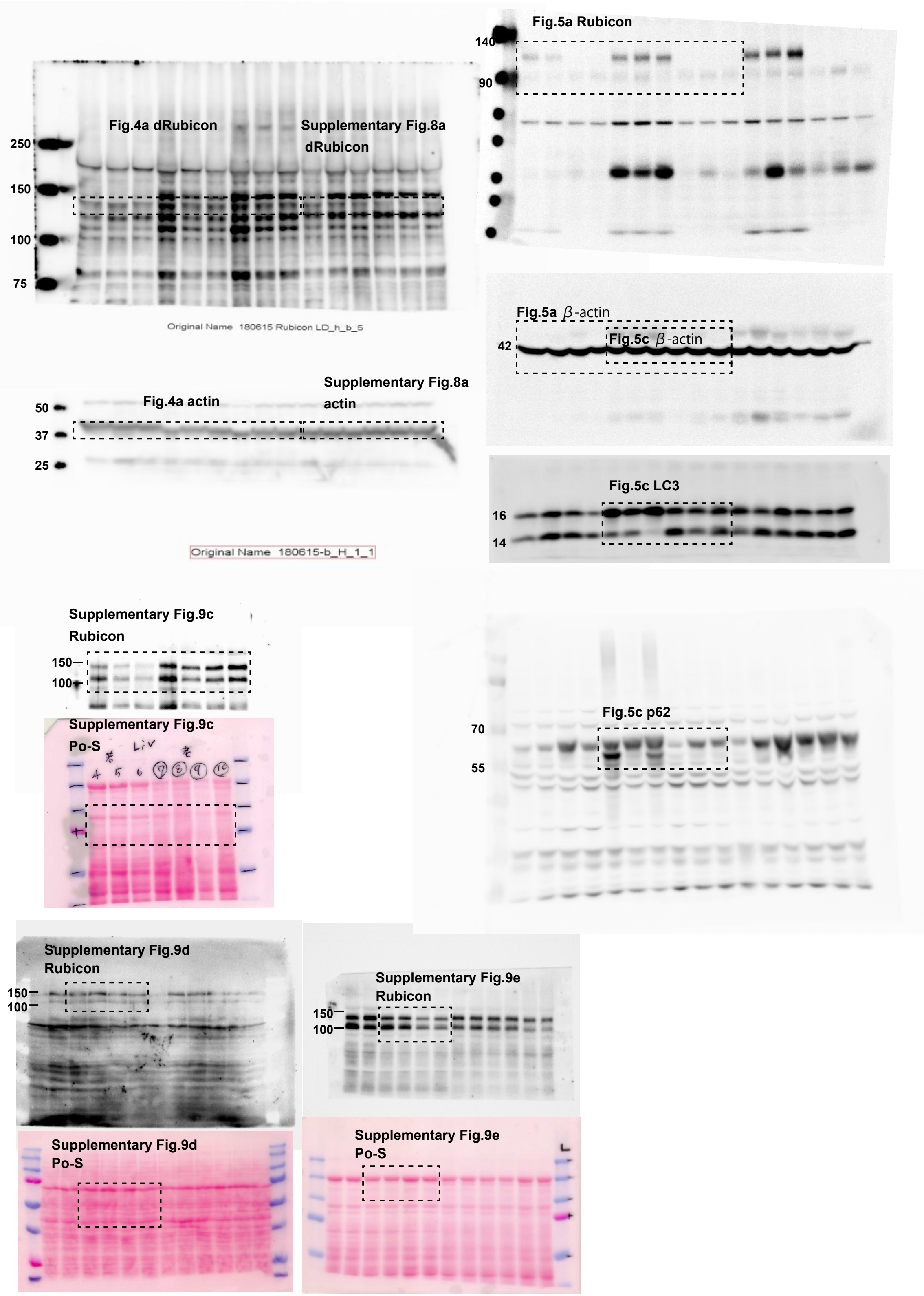

Supplementary Figure 10: Uncropped WB images

Supplementary Table 1: Sequence of qPCR primers

Worm
rub-1 forward 5′-TCACCAGTTCATGCTCTTCG-3′
rub-1 reverse 5′-CGTTCACGGAGTCTCCATTT-3′
atg-18 forward 5′-GAGGCGCGGCAAAAAGT-3′
atg-18 reverse 5′-CGTCCATATTCGGAACCGATT-3′
unc-51 forward 5′-AACGACAGGGATTTGTGGCA-3′
unc-51 reverse 5′- AAATGGGGGAAGAAGCAGCA-3′
bec-1 forward 5 - TGCAGTTGCTCGAGTTTTTG-3′
bec-1 reverse 5′- TGACACCATTGTCAACCAGTG-3′
ama-1 forward 5′-GGATGGAATGTGGGTTGAGA-3′
ama-1 reverse 5′-CGGATTCTTGAATTTCGCGC-3′
cdc-42 forward 5′-CTGCTGGACAGGAAGATTACG-3′
cdc-42 reverse 5′-CTCGGACATTCTCGAATGAAG-3′

Fly
dRubicon forward, 5′- CATGAATCGCTCCATCAAGG-3′
dRubicon reverse 5′- TGCAGTGCTCACAGACGAAG-3′
MJDtr forward, 5′ -GGGACCTATCAGGACAGAGT-3′
MJDtr reverse, 5′-TCAGATAAAGTGTGAAGGT-3′
Hsc70-1 forward 5′- TTTCTGTGCTGACCATCGAG-3′
Hsc70-1 reverse 5′- TGGCCCAGATCCTTCTTATG-3′

Mouse
Rubicon forward 5'-CTCATCCATGACCAGGTGTG-3'
Rubicon reverse 5'-GTCGCTCTCATGCAAACTGA-3'
GAPDH forward 5'-ATGGTGAAGGTCGGTGTGA-3'
GAPDH reverse 5'-AATCTCCACTTTGCCACTGC-3'
Col1a1 forward 5′- ACGCCATCAAGGTCTACTGC -3′
Col1a1 reverse 5′- ACTCGAACGGGAATCCATCG -3′
TGF-b1 forward 5′-ATGTCATGGATGGTGCCCAG-3′
TGF-b1 reverse 5′-GCTGAACCAAGGAGACGGAA-3′
18s rRNA forward 5′- AAACGGCTACCACATCCAAG-3′
18s rRNA reverse 5′- CCTCCAATGGATCCTCGTTA-3′

Supplementary Table 2: Worm strain lists

Strains	Genotype	Note
	N2 (WT)	
DA2123	*adIs2122 [lgg-1p::GFP::lgg-1 + rol-6(su1006)]*	
AM140	*rmIs132 [unc-54p::Q35::YFP]*	
TU3401	*sid-1(pk3321) V; uIs69 [pCFJ90 (myo-2p::mCherry) + unc-119p::sid-1]*	
VP303	*rde-1(ne219) V; kbIs7 [nhx-2p::rde-1 + rol-6(su1006)]*	
NR350	*rde-1(ne219) V; kzIs20 [hlh-1p::rde-1 + sur-5p::NLS::GFP]*	
NR222	*rde-1(ne219) V; kzIs9 [(pKK1260) lin-26p::NLS::GFP + (pKK1253) lin-26p::rde-1 + rol-6(su1006)]*;	
CB1370	*daf-2(e1370)III*	
DA465	*eat-2(ad465) II*	
CB4037	*glp-1(e2141ts)III*	
MQ887	*isp-1(qm150)IV*	
MAH215	*sqIs11 [lgg-1p::mCherry::GFP::lgg-1 + rol-6]*	
MAH44	*glp-1(e2141ts) III; adIs2122 [lgg-1p::GFP::lgg-1 + rol-6(su1006)]*	
MAH14	*daf-2(e1370) III; adIs2122 [lgg-1p::GFP::lgg-1 + rol-6(su1006)]*	
AA4045	*eat-2(ad465); adIs2122[lgg-1p::GFP::lgg-1 + rol-6(su1006)]*	
RT1970	*hTFR::GFP*	
SN7	*sqIs24 [rgef-1p::GFP::lgg-1 + unc-122p::RFP]; sid-1(pk3321) V; uIs69 [pCFJ90 (myo-2p::mCherry) + unc-119p::sid-1]*	Established in this work
SN2	*snEx2[rub-1p::rub-1::EGFP +myo-2p::mCherry]*	Established in this work
SN5	*snEx5[rub-1p::rub-1::EGFP +myo-2p::mCherry]*	Established in this work
