# Supplementary material for: Suppression of autophagic activity by Rubicon is a signature of aging
Source: Nat Commun. 2019 Feb 19;10:847. doi: 10.1038/s41467-019-08729-6 (PMC6381146; doi:10.1038/s41467-019-08729-6)
Supplement: Supplementary file 3 — Description of Additional Supplementary Files [file 41467_2019_8729_MOESM3_ESM.pdf]

Description of Additional Supplementary Files

File Name: Supplementary Data 1
Description: Detailed lifespan data with repeats and statistics.
