# Supplementary material for: Suppression of autophagic activity by Rubicon is a signature of aging
Source: Nat Commun. 2019 Feb 19;10:847. doi: 10.1038/s41467-019-08729-6 (PMC6381146; doi:10.1038/s41467-019-08729-6)
Supplement: Supplementary file 5 — Reporting Summary [file 41467_2019_8729_MOESM5_ESM.pdf]

nature research

Corresponding author(s): Tamotsu Yoshimori

Last updated by author(s): 2018/12/14

Reporting Summary

Nature Research wishes to improve the reproducibility of the work that we publish. This form provides structure for consistency and transparency in reporting. For further information on Nature Research policies, see Authors & Referees and the Editorial Policy Checklist.

Statistics

For all statistical analyses, confirm that the following items are present in the figure legend, table legend, main text, or Methods section.

n/a	Confirmed	
☐	☒	The exact sample size (n) for each experimental group/condition, given as a discrete number and unit of measurement
☐	☒	A statement on whether measurements were taken from distinct samples or whether the same sample was measured repeatedly
☐	☒	The statistical test(s) used AND whether they are one- or two-sided<br>*Only common tests should be described solely by name; describe more complex techniques in the Methods section.*
☒	☐	A description of all covariates tested
☒	☐	A description of any assumptions or corrections, such as tests of normality and adjustment for multiple comparisons
☐	☒	A full description of the statistical parameters including central tendency (e.g. means) or other basic estimates (e.g. regression coefficient) AND variation (e.g. standard deviation) or associated estimates of uncertainty (e.g. confidence intervals)
☐	☒	For null hypothesis testing, the test statistic (e.g. F, t, r) with confidence intervals, effect sizes, degrees of freedom and P value noted<br>*Give P values as exact values whenever suitable.*
☒	☐	For Bayesian analysis, information on the choice of priors and Markov chain Monte Carlo settings
☒	☐	For hierarchical and complex designs, identification of the appropriate level for tests and full reporting of outcomes
☒	☐	Estimates of effect sizes (e.g. Cohen's d, Pearson's r), indicating how they were calculated

Our web collection on statistics for biologists contains articles on many of the points above.

Software and code

Policy information about availability of computer code

Data collection	not applicable in this study
Data analysis	The ImageJ software was used to outline the puncta.<br>GraphPad Prism 7 was used to represent data in graphs and for the statistical analyses of the data.

For manuscripts utilizing custom algorithms or software that are central to the research but not yet described in published literature, software must be made available to editors/reviewers. We strongly encourage code deposition in a community repository (e.g. GitHub). See the Nature Research guidelines for submitting code & software for further information.

Data

Policy information about availability of data

All manuscripts must include a data availability statement. This statement should provide the following information, where applicable:

- Accession codes, unique identifiers, or web links for publicly available datasets
- A list of figures that have associated raw data
- A description of any restrictions on data availability

Gene accession codes were provided in the manuscript. Full scan of WB data were provided. All other data are available from the corresponding authors on reasonable request.

Field-specific reporting

Please select the one below that is the best fit for your research. If you are not sure, read the appropriate sections before making your selection.

☒ Life sciences ☐ Behavioural & social sciences ☐ Ecological, evolutionary & environmental sciences

For a reference copy of the document with all sections, see nature.com/documents/nr-reporting-summary-flat.pdf

Life sciences study design

All studies must disclose on these points even when the disclosure is negative.

Sample size	All experiments including qPCR, WB and lifespan are well established in worm, fly and mice. We have collected enough sample size based on the previous literature (Nakamura et al, 2016; Suzuki et al., 2015; Takahashi et al., 2017).
Data exclusions	For lifespan assays, worms that crawled off the plate or underwent vulva blasting could cause irregular death and were not included in the data. This exclusion criteria has been established and extensively used in the field.
Replication	All attempts at replication were successful.
Randomization	For lifespan and autophagy assay, worms and fly were chosen unbiasedly for experimental analysis to ensure randomization. For fibrosis analysis, several area in kidney were unbiasedly chosen for the quantification.
Blinding	Lifespan experiments were performed in a blinded manner.

Reporting for specific materials, systems and methods

We require information from authors about some types of materials, experimental systems and methods used in many studies. Here, indicate whether each material, system or method listed is relevant to your study. If you are not sure if a list item applies to your research, read the appropriate section before selecting a response.

Materials & experimental systems

n/a	Involved in the study	
☐	☒	Antibodies
☒	☐	Eukaryotic cell lines
☒	☐	Palaeontology
☐	☒	Animals and other organisms
☒	☐	Human research participants
☒	☐	Clinical data

Methods

n/a	Involved in the study	
☒	☐	ChIP-seq
☒	☐	Flow cytometry
☒	☐	MRI-based neuroimaging

Antibodies

Antibodies used	Primary antibodies and dilutions used for mouse tissues western blotting were as follows: for mice, Rubicon (Cell Signalling Technology, #8465, 1:500), LC3 (Cell Signalling Technology, #2755, 1:1000), p62 (MBL, PM045, 1:1000) and b-actin (Sigma Aldrich, A5316, 1:8000), for fly, anti-dRubicon (1:20,000), anti-actin (JLA20, 1:2,000, Developmental studies Hybridoma Bank). The specificity of dRubicon antibody was determined in Supplementary Fig.8a, b.
Validation	All antibodies validation are available on the manufacturers' websites or indicated above.

Animals and other organisms

Policy information about studies involving animals; ARRIVE guidelines recommended for reporting animal research

Laboratory animals	The following worm strains were used in the study; N2(WT); DA2123, adIs2122 [lgg-1p::GFP::lgg-1 + rol-6(su1006)] ; AM140, rmIs132 [unc-54p::Q35::YFP]; TU3401, sid-1(pk3321) V; uIs69 [pCFJ90 (myo-2p::mCherry) + unc-119p::sid-1]; VP303, rde-1(ne219) V; kbIs7 [nhx-2p::rde-1 + rol-6(su1006)]. NR350, rde-1(ne219) V; kzIs20 [hlh-1p::rde-1 + sur-5p::NLS::GFP]; NR222, rde-1(ne219) V; kzIs9 [(pKK1260) lin-26p::NLS::GFP + (pKK1253) lin-26p::rde-1 + rol-6(su1006)]; CB1370, daf-2(e1370)III; DA465, eat-2(ad465) II; CB4037, glp-1(e2141ts)III; MQ887, isp-1(qm150)IV; MAH215, sqIs11 [lgg-1p::mCherry::GFP::lgg-1 + rol-6]; MAH44, glp-1(e2141ts) III; adIs2122 [lgg-1p::GFP::lgg-1 + rol-6(su1006)]; MAH14, daf-2(e1370) III; adIs2122 [lgg-1p::GFP::lgg-1 + rol-6(su1006)]; eat-2(ad465); adIs2122[lgg-1p::GFP::lgg-1 + rol-6(su1006)]. MAH242, sqIs24 [rgef-1p::GFP::lgg-1 + unc-122p::RFP] was crossed with TU3401 to generate the neuron specific sensitive strain expressing neuron specific GFP::LGG-1. hTFR::GFP is a kind gift from Prof. Grant. Transgenic fly lines bearing UAS-MJDtrQ27 (#8149), UAS-MJDtrQ78s (#8150), UAS-MJDtr-Q78w (#8141), UAS-GFP-IR (#9330), UAS-dRubicon (CG12772)-IR (#43276), UAS-GFP-mCherry-Atg8a (#37749), da-GAL4 (#55849) and elav-GAL4c155 (#458) were obtained from the Bloomington Stock Center. Transgenic fly lines harbouring GMR-

GAL4 were described previously(Yamaguchi et al., 1999).CAG-Cre mice and Nestin-Cre mice were imported from Jackson Laboratory and Dr. Jun-ichi Miyazaki's laboratory (Osaka University), respectively. CAG-Cre mice were crossed with Rubiconflox mice(Tanaka eta al., 2016) to produce mice with systemic Rubicon deletion. Resultant mice with the Rubicon- allele were backcrossed into the C57BL/6J wild-type strain five times, followed by intercrossing between Rubicon+/- mice to generate Rubicon-/- mice and wild-type controls. The Nestin-Cre mice were crossed with Rubiconflox mice to produce mice harbouring homozygous deletion of Rubicon specifically in the brain. All mice used in this study were maintained on a C57BL/6J background, with the exception of the calorie restricted mice. For those experiments, we used an adult-onset 40% calorie restriction protocol developed by Turturro et al. Female BDF1 mice were reared individually in cages.

Wild animals	not applicable.
Field-collected samples	not applicable.
Ethics oversight	Experimental procedures using mice were approved by the Institutional Committee of Osaka University.

Note that full information on the approval of the study protocol must also be provided in the manuscript.
